# 4-Hydroxy-Trans-2-Nonenal in the Regulation of Anti-Oxidative and Pro-Inflammatory Signaling Pathways

**DOI:** 10.1155/2019/5937326

**Published:** 2019-11-06

**Authors:** Himangshu Sonowal, Kota V. Ramana

**Affiliations:** Department of Biochemistry and Molecular Biology, University of Texas Medical Branch, Galveston TX-77555, USA

## Abstract

Recent studies indicate that 4-hydroxy-trans-2-nonenal (HNE), a major oxidative stress triggered lipid peroxidation-derived aldehyde, plays a critical role in the pathophysiology of various human pathologies including metabolic syndrome, diabetes, cardiovascular, neurological, immunological, and age-related diseases and various types of cancer. HNE is the most abundant and toxic *α*, *β*-unsaturated aldehyde formed during the peroxidation of polyunsaturated fatty acids in a series of free radical-mediated reactions. The presence of an aldehyde group at C1, a double bond between C2 and C3 and a hydroxyl group at C4 makes HNE a highly reactive molecule. These strong reactive electrophilic groups favor the formation of HNE adducts with cellular macromolecules such as proteins and nucleic acids leading to the regulation of various cell signaling pathways and processes involved in cell proliferation, differentiation, and apoptosis. Many studies suggest that the cell-specific intracellular concentrations of HNE dictate the anti-oxidative and pro-inflammatory activities of this important molecule. In this review, we focused on how HNE could alter multiple anti-oxidative defense pathways and pro-inflammatory cytotoxic pathways by interacting with various cell-signaling intermediates.

## 1. Oxidative Stress and Lipid Peroxidation

**Figure 1:**
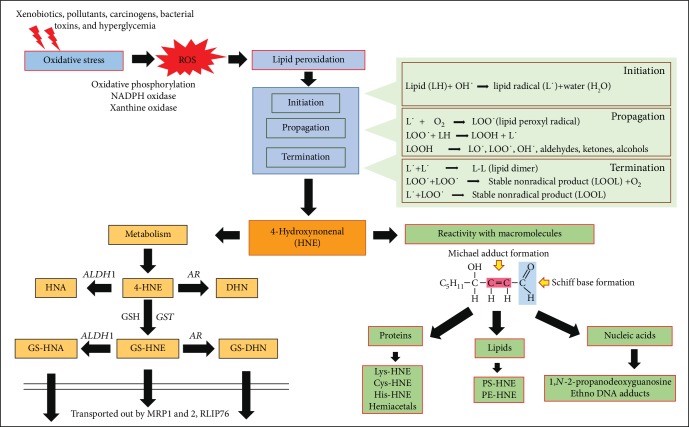
Schematic figure showing the ROS -induced formation of HNE via lipid peroxidation and its metabolism by various antioxidative enzymes.

Free radicals are regularly generated in aerobes because of normal respiration processes and the activity of cellular antioxidant defense machinery maintains a balance of the free radicals utilizing a variety of antioxidant enzymes in the cells. A balanced redox homeostasis is necessary for the maintenance of normal cellular processes in aerobes [1]. Under oxidative stress conditions, disruption of cellular redox homeostasis leads to an imbalance between reactive oxygen species (ROS) generation and their elimination by antioxidant enzymes. The major reasons for the redox imbalance could be the overproduction of free radical species or the inability of the cellular antioxidant defense machinery to eliminate or sequester the free radicals generated in the body. Free radicals such as superoxide anion (O_2_˙ˉ), hydroxyl radical (OH˙), nitric oxide (NO˙), peroxyl radical (LOO˙) and non-radical oxidants such as hydrogen peroxide (H_2_O_2_), peroxynitrite (ONOOˉ), hypochlorous acid (HOCl), nitrous acid (HNO_2_), and singlet oxygen (^1^O_2_), are the most commonly generated ROS in the cells and act as initiators of oxidative damage contributing to pathophysiology of multiple disease complications. Cells upon exposure to external oxidants such as xenobiotics, environmental pollutants, UV radiation, carcinogens and allergens, and internally formed oxidants in the body such as cytokines, growth factors, and chemokines could lead to altered cellular metabolic processes leading to the production of ROS. The activity of enzymes such as NADPH oxidase, xanthine oxidase, and auto-oxidation of glucose can generate ROS under different oxidant stimuli. In addition, mitochondrial oxidative phosphorylation that usually participates in the cellular respiration to generate energy in the cells can also contribute to the generation of free radicals (Figure 1). Apart from inducing damage to cellular macromolecules and dysregulation of cellular homeostasis, the free radicals formed during metabolic processes also act as secondary signaling intermediates and regulate various oxidative and anti-oxidative signaling pathways [2]. Antioxidant peptides such as glutathione (GSH) play an important role in detoxification of ROS. GSH is the most essential regulator of cellular redox homeostasis as it metabolizes or scavenges a number of free radicals or free radical- generated products in the cells. The ratio of GSH (reduced)/GSSG (oxidized) is a key indicator of cellular redox potential. Apart from GSH, enzymes such as glutathione-S-transferases (GSTs), plays a very important role in the maintenance of redox balance in cells. GSTs play a significant role in detoxification of various xenobiotics as well as endogenous toxic products generated in the cells by conjugation with GSH, facilitating further metabolism or detoxification by multiple other antioxidant detoxification and defense pathways [3]. Further, antioxidant enzyme superoxide dismutase (SOD) catalyzes the dismutation of superoxide free radical (O_2_˙ˉ), into hydrogen peroxide and oxygen. The hydrogen peroxide is further catalyzed by catalase into oxygen and water [4]. The activity of these antioxidant enzymes controls the free radical content in the body. However, during pathological complications, alterations in various antioxidant defense pathways along with increased ROS production imbalances cellular homeostasis leading to tissue damage and dysfunction.

Though ROS play an important role in regulating various physiological functions such as the elimination of pathogenic bacteria by immune cells, maintenance of vascular tone, cardiovascular functions, cell proliferation, and differentiation, a disturbance of ROS homeostasis leads to the development of various pathological complications. Several studies have shown that oxidative stress generated by ROS plays a critical role in multiple pathologies including various types of cancers, inflammatory disorders, neurodegenerative diseases, and cardiovascular complications [5, 6]. Apart from the spontaneous direct effect on DNA, RNA, amino acids, proteins and lipids, the secondary products generated by ROS -mediated reactions significantly damage various macromolecules and propagate their deleterious effects in the cells. Most importantly, even though the ROS generated in cells are short-lived, the secondary products generated by ROS are comparatively stable and further acts as important mediators of cellular signaling. The secondary ROS generated products maintain and propagate the effect of ROS long after ROS generation. These secondary toxic products could migrate to distant sites, which are far from their site of origin and can induce tissue damage and organ dysfunction in multiple sites, thus exerting multifactorial side effects. One such effect of ROS is the oxidative damage to the membrane lipids, which is termed as lipid peroxidation. Membrane phospholipids such as poly-unsaturated fatty acids (PUFA) are the major targets of lipid peroxidation induced by ROS. Lipid peroxidation-derived products such as HNE (4-hydroxy-trans-2-nonenal), acrolein and malondialdehyde (MDA) are more stable; have a longer half-life than ROS themselves and have attained considerable attention in the recent past as important mediators of oxidative stress-induced pathological complications [7].

**Figure 2:**
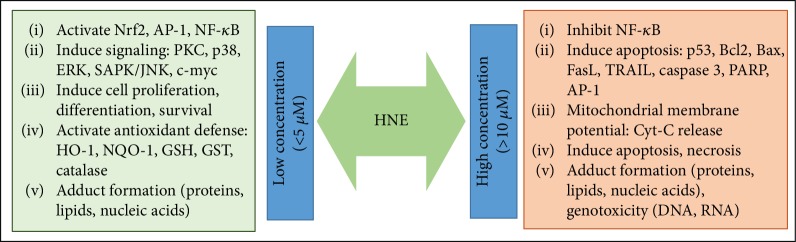
HNE is a pleiotropic signaling molecule: Depending on the concentration and duration of exposure, HNE induces multiple signaling pathways in cells by regulating various signaling intermediates.

During oxidative stress, lipid peroxidation occurs via three major steps, (a) initiation, (b) propagation and (c) termination. In the initial initiation step, the free radicals (e.g. OH˙) attack PUFA and generates lipid radicals (L˙). The removal of hydrogen atoms from the lipids leads to the reduction of ROS into water and generation of lipid radicals. During propagation step, the unstable lipid radicals react with oxygen leading to the generation of lipid peroxyl radicals (LOO˙) and additional lipid radicals (L˙), which further reacts in a chain reaction and new lipid radicals and lipid peroxides are generated. The presence of metal ions such as iron (Fe^2+^) and copper (Cu^2+^) is shown to accelerate the propagation reaction. In the final termination step, hydroperoxides are generated by the reaction of peroxyl radicals with vitamin E (*α*-tocopherol). The lipid peroxides and lipid radicals react with each other to generate more stable non-radical products during the termination of the lipid peroxidation process (Figure 1). The *β*-oxidation of lipid peroxides leads to the generation of various toxic lipid aldehydes such as alkanals, alkenals, hydroxyalkenals, and alkadienes [8, 9]. The formation of lipid aldehydes such as MDA and HNE is often considered as the most common toxic end products of lipid peroxidation and generally used as indicators of oxidative damage in the cells and tissues. Further, the *α*,*β*-unsaturated hydroxyalkenal, HNE has been shown to be the most abundant and toxic lipid peroxidation end product generated during lipid peroxidation [8, 9]. HNE with its highly reactive electrophilic groups can interact with cellular proteins, GSH, nucleic acids and can cause cytotoxicity or genotoxicity (Figure 1). Recent studies have postulated that HNE is a biomarker of oxidative stress-induced pathological complications and correlated its damaging effects to various human diseases such as cancer, neurodegenerative, inflammatory and autoimmune diseases, various metabolic diseases, and mitochondrial dysfunction [8, 10–15]. Besides its reactivity with macromolecules, HNE can also alter the membrane potential, electron transport and ion imbalance in the cells leading to neurological disorders [16, 17]. Further, HNE also plays an important role in cell survival and death by signaling through different pathways mediated by caspase3, Bax, Bcl2, death receptors, multiple kinases and transcription factors such as Nrf2, AP-1 and NF-*κ*B (Figure 2) [18–21]. The effect of HNE on the cellular macromolecules is dependent on the type of tissue and concentration of HNE, with specificities for proteins containing cysteine, histidine and lysine residues [8, 9, 22]. The detoxification and metabolism of HNE is also differential in different tissues [23]. In the succeeding section, we have discussed the reactivity of HNE with different macromolecules and its metabolism.

## 2. Reactivity and Metabolism of HNE

HNE has three functional groups 1) carbonyl group on C1 (C=O), 2) a double bond between C2 and C3 (C=C) and 3) hydroxyl group on C4 (which facilitates C=C polarization and cyclization reactions), which makes HNE a highly reactive aldehyde product of lipid peroxidation process (Figure 1). HNE can react with different macromolecules such as proteins, phospholipids, nucleic acids, and glutathione (GSH). Membrane-bound entities and proteins containing an abundance of cysteine, lysine and histidine residues and phospholipids such as phosphatidyl-ethanolamine are preferred targets for adduct formation by HNE. Electrophilic sites of HNE leads to Schiff base formation between an amino group and the carbonyl group at C1 and Michael addition of thiol or amino compounds at C3 [9, 24, 25]. The reactivity of HNE towards amino acids has been shown to be in the order of cysteine>histidine>lysine [26–28]. First, the amino acids undergo Michael addition at the C=C double bond of HNE. Michael addition to the C=C bond confers rotational freedom at the C2-C3 bond, which facilitates secondary reactions of primary amines with the carbonyl group of HNE to form Schiff bases [29]. Although cysteine is the most preferred amino acid for reactivity with HNE, HNE-histidine adducts have been shown to be more stable as compared to cysteine- and lysine-HNE adducts [28, 30]. Further, HNE-induced covalent modification of nucleophilic residues of amino acids regulate protein activation/inactivation, which could alter cellular signaling pathways. Specifically, important cellular signaling pathways controlling apoptosis, cell cycle, oxidative and nitrosative stress-associated pathways are reported to be significantly affected by HNE leading to cellular toxicities [31–33]. HNE also inhibits the proteasomes such as 20S proteasome and hence impairs the cellular proteasomal degradation of damaged or modified protein subunits generated subsequent to oxidative stress [34]. Chaperone activities specifically mediated by HSP 72 and HSP 90 have been shown to be modified by HNE adduct formation [35, 36]. HSP 90 function has been reported to be modified by the modification of Cys-572 residue by HNE, which has important pathological implications in alcoholic liver diseases (ALD) [36, 37]. HNE can directly interact with guanosine bases in DNA to form 1,N-2-propano-deoxyguanosine with an ability to form 1.2 ± 0.5 adducts/10^7^ nucleotides [38]. Etheno-DNA (ɛ-DNA) adducts have been shown to be generated by the reaction of HNE with nucleotides in DNA that could lead to mutational changes in DNA, increasing the susceptibility to cancerous transformation in cells [39]. Formation of HNE-DNA adducts indicate the genotoxic and mutagenic effects induced by lipid peroxidation [40]. HNE-DNA adduct formation hampers DNA repair mechanism in the cells. Studies have shown that HNE inhibits nucleotide excision repair in DNA damage-induced either by UV radiation or carcinogens such as benzo[a]pyrene diol epoxide (BDPE); a major environmental pollutant and component of cigarette smoke [41, 42]. Thus, these studies provide evidence that HNE is a multifactorial effector of signaling and cellular functions in the body.

HNE is short-lived in the cells with a half-life of less than 2 min and is immediately conjugated with other macromolecules or metabolized by various antioxidant enzymes [43]. Based on cellular antioxidant defense capacity and cell types, multiple studies have demonstrated varying concentrations and half-life of HNE in vitro and in vivo [44]. Major cellular metabolic pathways which play an important role in the detoxification of HNE are: (a) alcohol dehydrogenase (ADH) or aldose reductase (AR, AKR1B1), which reduces HNE to 1,4-dihydroxy-2-nonene (DHN), (b) aldehyde dehydrogenase (ALDH), which oxidizes HNE to 4-hydroxy-2-nonanoic acid (HNA) and (c) glutathione-S-transferases (GSTs), which catalyze the conjugation of HNE to GSH forming GS-HNE, which is then transported out of the cell in an ATP- dependent manner by various drug transporters such as MRP1, MRP2, and RLIP76 (Figure 1) [8, 45–47]. HNE has the ability to induce modifications in enzymes involved in cellular detoxification such as GSTs [48], glutathione reductase (GR) [49], either by Michael adduct formation or by covalent modifications of amino acid residues in the proteins. Interestingly, the cellular GSH levels control the concentration of HNE in cells and on the other hand, HNE has been shown to regulate the expression of enzymes responsible for GSH synthesis. Exposure of cells to HNE has been shown to increase *γ*-glutamate cysteine ligase (GCL) activity. GCL catalyzes the rate-limiting step in GSH biosynthesis and hence is important for the maintenance of cellular GSH levels. Further, HNE has also been shown to induce the transcriptional activation of *γ*-glutamyl cysteine synthase, thus contributing to enhanced GSH biosynthesis [50]. HNE can conjugate with GSH spontaneously or by the conjugation reactions catalyzed by the specific GST isozymes [51, 52]. GSTA4-4 and GST5.8 have been shown to specifically participate in the GSH-conjugation with HNE [53, 54]. Studies have shown that overexpression of HNE metabolizing GST (mGSTA4-4, hGSTA4-4, or hGST5.8) confers protection to cells against oxidative injury [55]. Exposure of hepatoma cells to exogenous HNE leads to the formation of major metabolites of HNE: glutathione-HNE (GS-HNE) indicating the importance of HNE-GSH conjugation as a major metabolic route for its detoxification [47]. Apart from GST, studies have also demonstrated that aldose reductase (AR; AKR1B1) further metabolizes GS-HNE to GS-DHN and aldehyde dehydrogenase (ALDH) isozymes metabolize GS-HNE to GS-HNA [54, 56]. Several studies have demonstrated that aldo-keto reductases are an important class of enzymes upregulated by oxidative stress or exposure to HNE [57]. Up-regulation of AR has been shown to play an important role in HNE detoxification in HepG2 cells. Burczynski et al., have demonstrated that HNE generated in the cells induces its own metabolism and detoxification by up-regulating the aldo-keto reductase isozyme AKR1C1 [58]. Both HNE and its GSH metabolite, GS-HNE, have been shown to be reduced by AR to 1,4-dihydroxy-2-nonene (DHN) and GS-DHN, respectively [59–62]. Further, AR reduces GSH conjugates of aldehydes (e.g. GS-HNE and GS-acrolein) more effectively as compared to their parent aldehydes (e.g. HNE and acrolein) indicating that AR has a specific binding site for glutathionylated aldehydes [63, 64]. Indeed, Singh et al., have crystallized AR bound GS-analogue and found that AR has specific GS-aldehyde binding domain [65]. These studies demonstrate that catalytic activity mediated by AR is a major step in the detoxification of lipid peroxidation-derived aldehydes such as HNE and their glutathione conjugates. Indeed multiple studies modulating AR activity have shown to exert therapeutic effects in various oxidative stress-induced inflammatory pathologies including cancer [56, 66–68].

HNE-mediated regulation of signaling pathways is pleiotropic and the signaling pathways activated or inhibited by HNE depends on the concentration of HNE and the type of cells used in the study. In cell culture studies, HNE concentration greater than 10 *μ*M has been reported to induce apoptosis whereas sub-lethal dose ≤5 *μ*M induces cell proliferation. Concentration-dependent activation of various pro-inflammatory and anti-oxidative pathways by HNE is shown to regulate multiple kinases and transcription factors important for disease pathology (Figure 2). In certain pathologies, a very high concentration of HNE has been observed. Elevated plasma levels of HNE (~100 *μ*M) have been reported in children with systemic lupus erythematosus [69] and in the liver tissues of mouse models of experimental alcoholic liver disease [70, 71]. However, it is still unclear how the cells cope with such high levels of HNE in vivo and the deleterious effect exerted by such high concentrations of HNE in various human diseases. With the increasing evidence of the importance of ROS and lipid peroxidation-derived aldehydes in different human pathologies, and advancement in analytical techniques to detect and identify the specific role of free radical intermediates in various diseases [72], studies on the roles of lipid peroxidation-derived aldehydes in modulating cellular signaling pathways has attained a considerable attention in the recent years. In the following section, we have briefed some of the important findings on the role of HNE in mediating anti - and pro-inflammatory signaling pathways.

## 3. Regulation of Anti-oxidative Pathways by HNE

**Figure 3:**
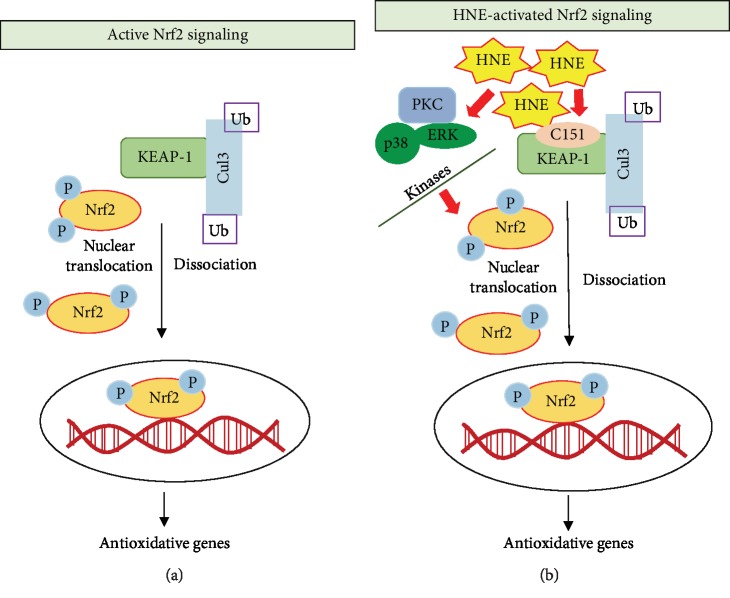
Role of HNE on Nrf2-mediated anti-oxidative signaling: Nrf2 is a master regulator of various anti-oxidative and anti-inflammatory pathways in the cells. Under basal conditions, Nrf2 is bound to KEAP-1 and remains inactive. KEAP-1 is an adaptor protein and association of KEAP-1 with Cul3, promotes proteasomal degradation of the Nrf2-KEAP1 complex. However, when activated by an external stimulus, (a) phosphorylation and dissociation of Nrf2 occurs from the Nrf2-Keap1 complex leading to nuclear translocation of Nrf2 and transcription of target genes. (b) HNE could promote Nrf2 activation either by modifying the C151 residues in KEAP-1 or by promoting Nrf2 phosphorylation directly. Apart from directly affecting Nrf2 and KEAP-1, various upstream kinases such as p38MAPK, PKC and ERK can also be modulated by HNE, which further initiates the activation of Nrf2.

NFE2-related nuclear factor 2 (Nrf2) is a master transcription factor regulating the expression of genes involved in the anti-oxidative and anti-inflammatory pathways [73]. Various exogenous stimuli such as xenobiotics, flavonoids, and antioxidants activate Nrf2 nuclear translocation, which then binds to its consensus antioxidant response elements (ARE) and induces the transcription of anti-oxidative genes. Under the basal conditions, Nrf2 is sequestered in the cytoplasm by its association with KEAP1, which keeps the complex inactive. The Nrf2-KEAP1 complex is ubiquitylated and subsequently, the association of Nrf2-KEAP1 complex with Cul3 induces proteasomal degradation. However, under conditions of oxidative stress or antioxidant stimuli, phosphorylation of Nrf2 triggers its dissociation from KEAP1. The active Nrf2 is then translocated to the nucleus and binds to its consensus ARE to activate respective antioxidant defense genes [74, 75]. (Figure 3).

Activation of the KEAP1-Nrf2 signaling pathway has been shown to be a major approach for HNE-induced cellular antioxidant defense. HNE is electrophilic in nature and hence activates the EpRE/ARE response elements. The highly reactive cysteine residues in KEAP1 makes it a preferred target for electrophilic attack by HNE [76]. Specifically, HNE has been shown to modify the cysteine amino acid residue at C151 in the BTB domain of KEAP1 leading to the dissociation of KEAP1 from the Nrf2-KEAP1 complex [76]. Three distinct cysteine amino acid-based sensors in KEAP1 have been reported to be recognized by carbonyl groups of HNE [77]. Apart from directly modulating KEAP1-Nrf2 interaction, HNE can also modulate various upstream protein kinases that are required for the phosphorylation Nrf2. Among these, PKC [78], PI3K [79] p38-MAPK, and ERK [80] have been shown to be important cellular protein kinases whose activities are specifically regulated by HNE. Although the precise mechanism of how these kinases modulate HNE-induced Nrf2 activation is not known, it is hypothesized that the adduct formation of protein kinases with HNE could alter the physiological functions of these protein kinases and favor phosphorylation of Nrf2 at specific sites (Figure 3).

Activation of Nrf2 either directly or indirectly acts as an important transcriptional regulator of various Phase -II detoxifying enzymes conferring protection to cells against oxidative damage [81]. Some important Phase- II detoxification enzymes activated by HNE include aldo-keto reductases (AKRs), *γ*-glutamylcysteine ligase (GCL), glutathione peroxidase (GPX), glutathione-S-transferase (GST), NADPH quinone oxidoreductase 1 (NQO1), heme oxygenase 1 (HO-1), thioredoxin (Trx), thioredoxin reductase (TrxR), and drug transporter proteins such as MRPs, Pgp1 and RLIP76. All the Phase-II detoxification enzymes have been shown to have specific ARE consensus sequences for Nrf2 binding in response to oxidative stress [82]. It is important that under oxidative stress, the major cellular detoxification pathways are activated in a concerted mechanism as a defense response to maintain the cellular redox balance homeostasis to confer protection against oxidative injury.

Several studies have demonstrated that the activation of Nrf2 plays an important role in the detoxification of xenobiotics, including lipid peroxidation products such as HNE [83, 84]. Miller et al., have shown that the intraperitoneal injection of Nrf2-ARE activators such as sulforaphane or carnosic acid protects cerebral cortical mitochondria from HNE-induced toxicity. Further, treatment with sulforaphane or carnosic acid up-regulated the mRNA levels of antioxidant enzymes such as HO-1 and prevented HNE-induced disruption of mitochondrial respiration [85]. HNE has also been shown to induce the expression of antioxidant enzymes AKR1C1, GSTA4, and HO-1 in HeLa cells, which play an important role in the protection against oxidative stress-induced toxicity [86]. Using siRNA-mediated silencing of Nrf2, this study has provided strong evidence that Nrf2 plays an important role in exerting protection against HNE-induced toxicity in HeLa cells [86]. Exposure to low concentrations of HNE (<5 *μ*M) is likely to induce an elevated anti-oxidant response, which prepares the cells to cope with oxidative insults. Incubation of macrophages and vascular smooth muscle cells with HNE has been shown to activate Nrf2 and protect cells from oxidative injury during vascular complications. Abundant formation of lipid peroxidation products such as HNE has been reported in atherosclerotic plaques and hence identifying the signaling pathways regulated by HNE in vascular cells is of immense importance in atherosclerosis [87]. Gargiulo et al., have shown that the exposure of monocytes to HNE induces various inflammatory cytokines such as IL-1*β*, IL-8, and TNF-*α* which regulate atherosclerotic plaque stability [88, 89]. Incubation of HNE with murine peritoneal macrophages isolated from Nrf2 knockout mouse models did not show increased expression of HO-1, Prx1, and A170 antioxidant proteins, indicating the significance of Nrf2 in HNE-induced protective effects [88]. HNE-induced upregulation of CD36, a major scavenger receptor, has been shown to exert protective functions during atherosclerosis and oxLDL-induced oxidative damage in an Nrf2 dependent manner [90]. Studies have demonstrated that multiple antioxidant response elements and oxLDL uptake receptors are HNE targets, which play an important role in atherosclerosis progression, the anti-oxidant response in endothelial and vascular cells and atheroprotective functions [91, 92]. In 661 W retinal ganglion cells, apart from upregulating the nuclear translocation and Nrf2-ARE transcription activity, treatment with 5 *μ*M HNE induced the expression of antioxidant genes such as Trx, TrxR, and HO-1, exerting protective functions. Similarly, the up-regulation of antioxidant genes by HNE conferred protection against H_2_O_2_-induced apoptosis in 661 W cells [93]. This study also demonstrated that siRNA-mediated silencing of Nrf2 failed to confer protection induced by HNE against H_2_O_2_-induced cell damage [93]. However, the signaling mechanisms activated by HNE are elusive. As we have already discussed in the preceding section that the concentration of HNE used in a study could act as an important factor to properly decipher the signaling mechanisms activated or inhibited by HNE (Figure 2). In studies using primary cultures of human optic nerve head astrocytes, it is observed that HNE concentrations greater than 50 *μ*M induce apoptosis and significantly decrease the cellular GSH levels [94]. Treatment with a lesser concentration of HNE (< 25 *μ*M) also leads to a significant decrease in cellular GSH levels after 1 h and 3 h of treatment. However, when the cells were allowed to recover for 24 h without HNE after 1 h or 3 h treatment with 25 *μ*M HNE, significant restoration of GSH levels have been observed [94]. In this study, real-time PCR gene expression analysis of mRNA also showed a significant increase in the levels of AKR1C1, GSTA4 and GCLC mRNA along-with increase in transcription factor Nrf2 and c-Fos. Thus, the concentration and time of exposure to HNE are important in mediating HNE-induced antioxidant responses, which prepares the cells for protection against oxidant-induced damage [94]. It is already clear that strategies such as using natural or synthesized antioxidants to activate or augment Nrf2 mediated cellular antioxidant defense pathways exert protective functions against oxidative stress injury by multiple oxidants including lipid peroxidation products [86, 95]. Thus, HNE-mediated activation of Nrf2 may be an important step in regulating the HNE-induced antioxidant response in cells and tissues.

Studies to decipher the molecular mechanisms of HNE-mediated antioxidant defense response has provided evidence that apart from Nrf2-mediated regulation of antioxidant response, HNE has the ability to directly regulate the transcriptional activity of other antioxidant enzymes responsible for GSH biosynthesis. HNE regulates GSH levels by regulating the rate-limiting step of GSH biosynthesis by modifying Cys553 of the catalytic subunit (~73 kDa GCLC) and Cys35 of the modulatory subunit (~31 kDa, GCLM) of glutamate-cysteine ligase (GCL) [96]. GCL is a heterodimeric holoenzyme complex consisting of a 73 kDa catalytic subunit (GCLC) and a 31-kDa modifier subunit, which catalyzes the first and rate-limiting step of GSH biosynthesis [97]. Further, HNE induces the promoter activity of *γ*-glutamyl transpeptidase (GGT) by interacting with an electrophile response element (EpRe) in the proximal region of GGT promoter region 5 (GP5). GGT plays an important role in glutathione homeostasis and metabolism of GSH and hence increasing the transcriptional activity of GGT confers protection against oxidative injury. Moreover, HNE has been shown to be involved in Nrf2 binding to the GP5 EpRE complex [98]. Apart from directly inducing GGT promoter activity, HNE could also activate various upstream MAPK kinases such as ERK and p38-MAPK, which are involved in the activation of GGT. The transcriptional regulation of EpRE by p38-MAPK and ERK has been shown to induce GGT mRNA transcription, which regulates antioxidant response and cellular homeostasis [99]. MAPKs could act as important components in the regulation of Nrf2 activity induced by HNE as they have been reported to directly induce phosphorylation of Nrf2 leading to its activation [80]. Further, the transcription factor AP1 has been shown to be involved in the HNE-induced increase in the glutamate cysteine ligase (GCL) expression by regulating antioxidant response [100, 101]. These studies have provided evidence that multiple signaling pathways co-operate to induce antioxidant defense response induced by HNE. Low concentrations of HNE has been shown to induce the expression of cytoprotective genes such as thioredoxin reductase 1 (TrxR1) in a Nrf2/ERK/Akt-dependent pathway in PC12 cells [102]. Further, the expression of TrxR1 has been shown to induce an adaptive response and enhances cellular tolerance to oxidative insult. The HNE-induced ERK and AKT signaling pathways may directly or indirectly affect Nrf2 expression in PC12 cells [102]. HNE-mediated activation of ERK also plays an important role in the regulation of HO-1 expression induced by HNE. In rat pulmonary epithelial cells, HO-1 expression is reported to be translationally regulated by HNE-mediated phosphorylation of ERK [103].

ROS are important for HNE production; ROS-induced lipid peroxidation is a major source of HNE generation in cells [7, 9]. Furthermore, studies have provided evidence that HNE itself could induce ROS generation in cells; the HNE-induced ROS further propagates the effects of HNE-mediated antioxidant defense mechanisms in cells [104]. On the other hand, a few studies showed that ROS could play a bystander effect on HNE-induced cell signaling. Studies on PC12 cell lines provide evidence that HNE-induced cell damage was a result of HNE-induced modification of cellular GSH or modification in cysteine and histidine residues in proteins rather than the direct effect of ROS alone. HNE-induced apoptosis, intercellular Ca^+2^ accumulation, caspase activation and cellular quiescence were not ameliorated by scavenging the ROS generated in cells [105]. Thus, protein modifications, rather than ROS generation in HNE exposed cells seem to be a major regulator of HNE-mediated cell signaling [105]. Several other pathways such as the unfolded protein response- (UPR) and ER stress-associated signaling pathways have been shown to be affected by HNE [105]. In the same study, they have demonstrated that HNE induces ER stress by regulating ATF4 expression which regulates the unfolded protein response (UPR). A significant increase in UPR target genes such as GRP78 and CHOP has been observed in PC12 cells treated with HNE [105]. Thus, HNE by interacting with various protein kinases such as MAPK and AKT, transcription factors and AREs regulates the Nrf2-mediated antioxidant pathways in the cells. Furthermore, exposure of cells to low concentrations of HNE prepares the cells to withstand further oxidative injury, whereas a high concentration activates the apoptotic pathways leading to cellular toxicity.

## 4. Regulation of Pro-Inflammatory Pathways by HNE

Besides the regulation of Nrf2-mediated anti-oxidant pathways, HNE has been shown to regulate various pro-inflammatory pathways mediated by NF-*κ*B and AP1. The activation of these pathways leads to the expression of multiple cytokines, chemokines and growth factors responsible for inflammatory response and various disease pathologies. NF-*κ*B; a major redox-sensitive transcription factor activated during oxidative stress conditions, is a key regulator of cell viability and death [106]. Various studies have provided evidence of the myriad and elusive role of HNE as a critical regulator of NF-*κ*B-mediated inflammatory signaling pathways in cells. NF-*κ*B is a heterodimeric transcription factor with five subunits consisting of p50, p52, p65, c-Rel, and RelB. In the inactive state, the NF-*κ*B complex is localized in the cytoplasm due to its association with I*κ*B*α*. Upon stimulation by exogenous stimuli, NF-*κ*B is phosphorylated and translocated to the nucleus where it binds to its consensus DNA sequence element and induces transcription of target genes. The I*κ*B kinases (IKK) play an important role in the activation of NF-*κ*B by inducing phosphorylation on serine residues of I*κ*B*α*, which leads to dissociation of I*κ*B*α* from NF-*κ*B, leading to NF-*κ*B activation and nuclear translocation. After dissociation, I*κ*B*α* is polyubiquitinylated and degraded by the proteasome [106].

**Figure 4:**
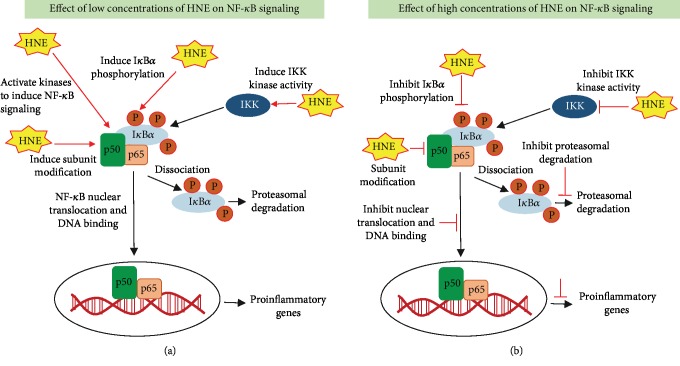
Role of HNE on NF-*κ*B-mediated pro-inflammatory signaling: During oxidative stress conditions ROS activate a series of protein kinases such as IKK which phosphorylates IkB*α* leading to dissociation of the inactive complex of NF-*κ*B-I*κ*B*α*. The active NF-*κ*B is translocated to the nucleus and binds to its consensus sequences to induce the transcription of target genes. HNE exerts pleiotropic effects on NF-*κ*B; (a) the low concentrations of HNE (<5 *μ*M) could activate NF-*κ*B whereas (b) high concentrations (>5 *μ*M) of HNE could inhibit NF-*κ*B. HNE could modulate NF-*κ*B activity by interacting with upstream protein kinases and can directly modify NF-*κ*B subunits.

HNE can induce either activation or inhibition of NF-*κ*B depending on the cell type and concentration (Figure 4). Although the mechanisms are not clearly understood, it has been shown in many studies that exposure to a high concentration of HNE inhibits the activation of NF-*κ*B [107]. In fact, oxidative stress-induced HNE and NF-*κ*B activation are positively correlated with the increased inflammatory response in many studies [108–113].

It has been demonstrated in THP1 cells that HNE prevents phosphorylation of I*κ*B*α*, which is necessary for NF-*κ*B activation. Specifically, pre-treatment of THP1 cells with 25 *μ*M HNE resulted in the reduction of LPS-induced phosphorylation in Ser-32 of I*κ*B*α*, thus preventing the activation of NF-*κ*B by HNE [114]. Besides LPS, HNE also inhibits phosphorylation of I*κ*B*α* induced by IL-1*β* and Phorbol 12-myristate 13-acetate (PMA) in monocytes [114]. Studies have further demonstrated that HNE could directly regulate the activity of IKK Kinase by forming covalent adducts with the IKK complex. IKK-induced phosphorylation I*κ*B*α* is necessary for NF-*κ*B activation [115]. Studies using H1299 and Jurkat T-cells have shown that pre-treatment with HNE blocks TPA- or ionomycin-induced activation of NF-*κ*B [115]. HNE could also regulate NF-*κ*B by directly forming adducts with important signaling intermediates. In a model of long-term hepatic injury following alcohol exposure to rodents, HNE-I*κ*B*α* adduct formation has been demonstrated to inhibit NF-*κ*B, which is independent of IKK phosphorylation [116]. Inhibition of NF-*κ*B by HNE exerts anti-inflammatory effects. HNE-induced inhibition of NF-*κ*B prevents LPS-induced production of IL-6 in rat kupffer cells [117]. HNE-IKK adduct formation and suppression of I*κ*B*α* phosphorylation could lead to the inhibition of NF-*κ*B. HNE has been shown to prevent IL-1*β*-induced nuclear translocation of p65 and NF-*κ*B-DNA binding [118]. Similarly, in rat cortical neuronal cells, exposure to HNE prevented the NF-*κ*B-DNA binding activity [119].

On the other hand, many studies have provided substantial evidence that HNE activates NF-*κ*B (Figure 4). Exposure of cells to low concentrations of HNE (1 *μ*M) induces the activation of NF-*κ*B in vascular smooth muscle cells [120]. The same concentration of HNE has been shown to induce phosphorylation and DNA-binding of NF-*κ*B [120]. Similarly, another study demonstrates that exposure of vascular smooth muscle cells to HNE increases ROS production and activation of NF-*κ*B/AKT signaling pathway leading to cell proliferation [121]. 5-Lipoxygenase (5-LOX) plays an important role in inflammation and atherosclerotic plaque formation. HNE has been shown to induce 5-LOX mRNA expression in murine macrophages by signaling through NF-*κ*B, p38MAPK and ERK [122]. Similarly, exposure of murine macrophages to LPS increased ROS production and subsequently increased lipid peroxidation products [123]. Results from this study also demonstrate that HNE is an important mediator of LPS-induced inflammation by increasing the release of inflammatory cytokines such as TNF-*α*, IL-1*β*, IL-6, and MCP-1 [123]. HNE-mediated increase in the NF-*κ*B activity has been shown to be involved in inflammatory signaling in rheumatoid arthritis. Treatment of synovial cells with 5 *μ*M HNE resulted in a time-dependent increase in IL-1*β*, IL-6, and TNF*α* [124]. Exposure to a higher concentration of HNE (50 *μ*M), led to a transient increase in IL-1*β*, IL-6, and TNF-*α*, with the maximum increase observed after 1 h. However, the levels of inflammatory cytokines gradually decreased after 6 and 12 h. Increase in Cox2 expression, an important inflammatory mediator was also observed in the same study [124]. Further, HNE has been reported to induce endothelial dysfunction, by increasing the production of NF-*κ*B-mediated expression of IL-8, ICAM, and impairment of endothelial barrier function [125].

Although the literature provides conflicting data on the role of HNE in inhibiting or activating NF-*κ*B based on its concentration, it is clear that exposure of cells to HNE induces a state of oxidative stress imbalance in the cells. Exposure to HNE leads to depletion of cellular GSH [126] and studies have provided evidence that depletion of GSH leads to activation of NF-*κ*B [127]. HNE has also been reported to induce p47phox-mediated NADPH oxidase activity in murine macrophages contributing to oxidative stress [128]. Although the involvement of HNE-induced ROS in HNE-mediated signaling is controversial, studies have shown that HNE-induced mitochondrial damage in cells is primarily due to ROS-generation, which is also a major contributor of vascular damage induced by HNE [129]. HNE-induced oxidative stress has been shown to be an inducer of endothelial dysfunction. Several studies have demonstrated that treatment with antioxidants such as NAC and mercaptopropionyl glycine, which inhibits oxidative stress, attenuated the HNE-induced loss of endothelial cell function [130, 131]. These studies highlight that HNE-induced oxidative stress as a major player in HNE-induced endothelial cell damage and dysfunction.

Apart from directly regulating NF-*κ*B, multiple lines of evidence suggest that HNE induces NF-*κ*B -mediated pro-inflammatory signaling by regulating the activation of major protein kinases such as PKC, p38-MAPK, and JNK [132]. PKC is an important kinase in mediating HNE signaling. Treatment of RAW 264.7 macrophages with HNE decreases phorbol 12-myristate 13-acetate (PMA)-induced ROS production in a dose- and time-dependent manner by interacting with PKC [133]. Further, MAPK kinases are also reported to play an important role in HNE-induced endothelial dysfunction. In a study using human macrophages, induction of Cox2 expression was observed upon exposure to HNE. In this study, the authors did not observe any change in the expression of iNOS or NF-*κ*B but p38-MAPK was found to be an important regulator of HNE-induced inflammatory signaling in macrophages [87]. HNE also alters the balance of calcium ions in cells, which is important for signal transduction mediated by PKC and PI3K isozymes. In human chondrocytes, treatment with HNE induced the HNE-IKK adduct formation leading to inhibition of nuclear translocation of p65 and DNA-binding. Surprisingly, data from the same study show that HNE induces Cox2 and PGE2 in chondrocytes independent of NF-*κ*B [118].

Activator protein 1 (AP-1) transcription factor is an important regulator of cellular signaling pathways controlling differentiation, proliferation, and apoptosis. HNE is well known to alter AP-1 transcriptional activity and affects various cellular processes. In rat cortical neurons, HNE-induced AP-1 nuclear translocation and DNA-binding plays an important role in neuronal calcium homeostasis and mitochondrial dysfunction [119]. Furthermore, the regulation of AP-1 activity by HNE has been shown to induce vascular smooth muscle cell proliferation leading to vascular complications [134].

HNE has been shown to be involved in the regulation of both intrinsic and extrinsic apoptotic signaling pathways in cells [19]. Exposure of SK-N-BE neuroblastoma cell lines to HNE induces the expression of p53, p73, p63, p21 and Bax leading to apoptotic cell death [135]. HNE has also been reported to induce p53-mediated apoptosis in the retinal pigment epithelial cells. Using knockout mouse models of GSTA4-4, Sharma et al., have demonstrated that HNE induces p53-mediated apoptotic signals via p21, Bax, and caspase 3. Inhibiting the cells ability to detoxify HNE leads to HNE-induced phosphorylation and nuclear translocation of p53 and induction of apoptosis [136]. In addition to HNE-induced p53 expression, HNE-mediated cytochrome C release has been shown to be necessary for HNE-induced apoptosis in macrophages [137].

HNE also induces the activation and phosphorylation of Src kinases. Mass spectrophotometric analysis of HNE-Src interaction showed that HNE interacts with His236, Cys241 and Cys248 residues of Src. Activation of Src by HNE leads to the expression of inflammatory mediator Cox2 and transcription factor AP-1, via activation of p38MAPK, JNK and ERK1/2 [138]. The HNE-Src adduct formation plays an important role in pro-inflammatory signaling in aged kidneys [139]. HNE also plays an important role in age-related oxidative stress. Higher plasma levels of HNE has been reported in obese individuals [140]. HNE-induced TNF-*α* expression in adipocytes was regulated by HNE mediated transcriptional regulation of ETS1 transcription factors [140]. HNE induced inflammation and Cox-2 production in many studies has been reported to be mediated by p38-MAPK mediated signaling and activation of ATF-2/CREB-1, JNK, c-JUN and AP-1 [20]. Many other transcription factors have been reported to play an important role in HNE-induced vascular endothelial cell dysfunction [141]. Transcription factors such as ATF3 and ATF4, have been reported to play an important role in HNE- induced ER stress in endothelial cells. The siRNA mediated ATF4 deletion prevented HNE-induced monocyte adhesion and IL-8 production and exerted protective functions against HNE-induced toxicity in endothelial cells [126].

**Figure 5:**
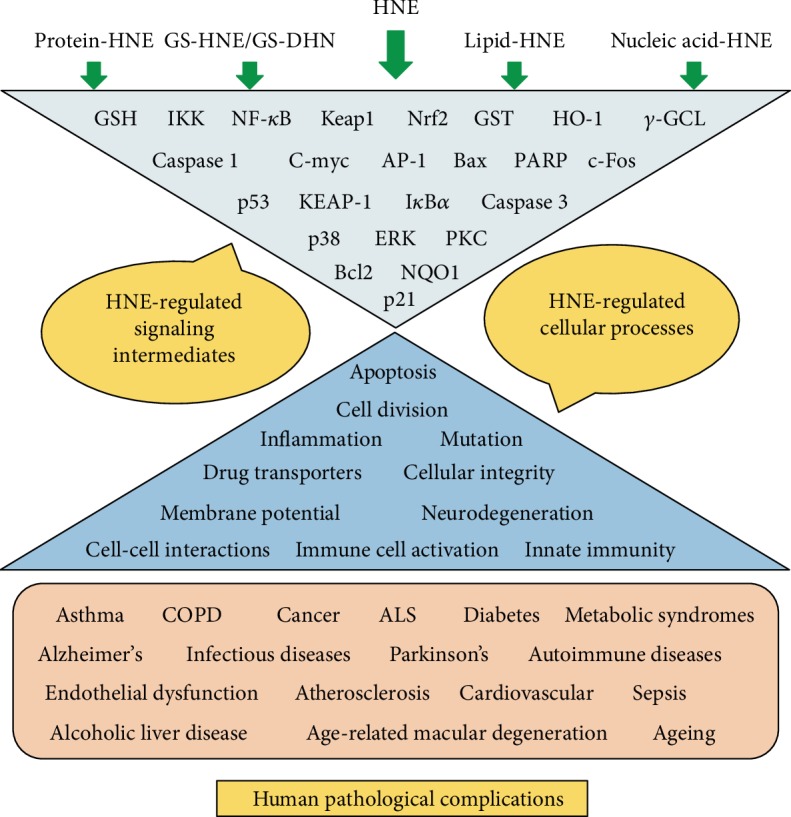
Central role of HNE and its metabolites in the regulation of various signaling pathways leading to various human disease pathologies. A summary of important signaling intermediates and cellular processes affect by HNE and its metabolites which can contribute to the pathophysiology of various human disorders are listed.

HNE also plays an important role in cancer-associated inflammation and cancerous progression as evidenced by recent reports highlighting the importance of HNE-mediated pro-inflammatory signaling in cancer [142]. HNE is an important second messenger molecule modulating cellular signaling pathways in cancer and mediates cancer cell proliferation, apoptosis, and antioxidant defense [44]. HNE either by extrinsic or intrinsic mechanisms has been reported to facilitate cancerous progression [9, 13, 41, 44]. Co-administration of HNE accelerated DSS-induced colitis in mice. Along with the increase in DSS-induced colitis, a significant increase in the expression of pro-inflammatory genes such as IL-6, TLR4, Cox2 along-with infiltration of CD45^+^ and CD45^+^F4/80^+^ immune cells were observed in HNE + DSS treated mice compared to DSS-alone. Treatment with HNE also impairs intestinal barrier function by reducing the colonic expression of occludin protein. Loss of barrier function leads to an increase in the bacterial LPS in the circulation. Further, by using TLR4 null mouse models they have demonstrated that HNE mediates inflammatory signaling in DSS-induced colitis by signaling through TLR4 [143]. Apart from this, analyzing transcript levels of genes altered by HNE in human colorectal carcinoma cells have provided evidence that exposure to HNE induces an alteration in multiple signaling pathways related to antioxidant response, ER stress, apoptosis and cell cycle regulation in a time- and dose-dependent manner [144]. Apart from cancer progression, studies also provide evidence that HNE plays an important role in inducing apoptosis in colon carcinoma cells. It has been demonstrated that HNE-induced apoptosis in colon carcinoma cells is mediated by signaling through HSF-1. siRNA-mediated inhibition of HSF-1 prevented HNE-induced cleavage of PARP and caspase 3. Overexpression of Bcl-xl attenuated HNE-induced apoptosis in colon carcinoma cells [145]. HNE also plays an central role in the pathogenesis of inflammatory lung pathologies such as COPD. High levels of HNE-modified proteins have been observed in lung tissues from COPD patients with a positive correlation to inflammation and expression of inflammatory mediators [146]. Recent studies have also demonstrated the importance of HNE in neuronal inflammation and maintenance of neuronal cells. Studies using animal models suggest that HNE reduces neuronal intracellular calcium (Ca^+2^) in CSF and leads to the loss of motor neurons. Calcium levels were also altered in the surviving neurons with no observable morphological alterations. Thus, HNE induces a prominent change in ionic equilibrium in neuronal cells implicating its importance in neurodegenerative diseases [147, 148]. The importance of HNE and lipid peroxidation products in neuronal biology and neuroinflammation is attaining considerable attention in recent years [148, 149]. HNE induces the expression of Cox2, PGE2 and IL-6 expression and inhibition of HNE is a beneficial target to attenuate inflammation during osteoarthritis [150, 151]. Apart from the evidence provided above on the role of HNE in inflammatory pathologies, HNE also plays an important role in the pathogenesis and progression of other inflammatory diseases such as cataract, AMD and COPD [152]. Thus, these studies provide evidence that HNE is an important mediator of various pro-and anti-inflammatory signaling pathways in different cell types (Figure 5) and understanding the cellular signaling pathways regulated by lipid peroxidation-derived aldehydes such as HNE will provide insights into the mechanism of disease and open avenues for the development of new therapeutic strategies in the future.

## 5. Conclusions and Future Perspectives

Recent studies have shown that depending upon the cell type, concentration and adduct formation with macromolecules, HNE could dictate cells to undergo proliferation or death. Interaction of HNE with cellular GSH has been shown to be a major metabolic route for its detoxification as well as intervening the cellular signaling pathways. Recent studies also suggest that HNE and HNE-protein adducts could act as biomarkers of various disease processes. Importantly, HNE metabolizing enzymes such as AR, ALDH1, and GSTs could regulate various anti-oxidative and pro-inflammatory pathways by generating HNE metabolites such as GS-HNE and GS-DHN which can further act as secondary signaling molecules. These studies opened new dimensions to understand the significance of HNE as well as other lipid aldehydes formed during lipid peroxidation and to understand the significance of aldehyde metabolizing enzymes in the pathophysiology of various diseases. These studies have provided a redox link between lipid aldehyde formation with oxidative stress and immune and inflammatory responses. Most importantly, we have shown that AR that reduces HNE, acrolein and other lipid aldehydes mediate oxidative stress signals initiated by various oxidants such as allergens, bacterial toxins, hyperglycemia, cytokines, and growth factors [56]. In fact, inhibition of AR has been shown to prevent several inflammatory complications including colon cancer, asthma, sepsis, uveitis, and cardiomyopathy [67, 68]. Thus, the specific use of antioxidants or synthetic inhibitors of HNE metabolizing enzymes that control the intracellular levels of HNE and its GSH-metabolites could be developed as potential therapeutic drug targets. It is a challenge for future research to clearly understand the complex interactions of HNE with various cellular proteins and how the protein-HNE adducts regulate various physiological processes. Further, it is still not clear how HNE commands cell signaling pathways based on a specific cell or tissue type. A better understanding of HNE-adduct formation with cellular macromolecules and identification of new HNE-adducts as potential biomarkers for various human pathologies will help to understand the pathophysiology of disease progression. In addition, identification of new immune and inflammatory response pathways regulated by HNE and its metabolites could help to understand the tissue and organ damage and dysfunction. Further, the use of novel transgenic approaches, metabolomics and next-generation sequencing (NGS) could ease the identification of critical signaling pathways intervened by HNE and its metabolites.

## Abbreviations

ADH: Alcohol dehydrogenase
AKR1B1: Aldo-keto reductase family 1 member B1
ALD: Alcoholic liver disease
ALDH: Aldehyde dehydrogenase
AMD: Age-related macular degeneration
AP-1: Activator protein-1
AR: Aldose reductase
ARE: Antioxidant response element
ATF4: Activating transcription factor 4
COPD: Chronic obstructive pulmonary disease
Cox-2: Cyclooxygenase 2
CREB: cAMP response element-binding protein
CSF: Cerebrospinal fluid
Cul3: Cullin3
DHN: 1,4-dihydroxy-2-nonene
DNA: Deoxyribonucleic acid
DSS: Dextran sulfate sodium
EpRE: Electrophile responsive element
ERK: Extracellular signal-regulated kinase
ETS1: ETS proto-oncogene1
GGT: *γ*-Glutamyl transpeptidase
GPX: Glutathione peroxidase
GRP-78: Glucose regulated protein 78
GSH: Glutathione
GSSG: Glutathione disulfide
GST: Glutathione-S-transferase
H_2_O_2_: Hydrogen peroxide
HNA: 4-hydroxy-2-nonanoic acid
HNE: 4-hydroxy-trans-2-nonenal
HO-1: Heme oxygenase-1
HSF-1: Heat shock factor-1
ICAM-1: Intercellular adhesion molecule -1
IKK: I-kappaB kinase
IL-6: Interleukin-6
iNOS: Inducible nitric oxide synthase
I*κ*B*α*: Nuclear factor of kappa light polypeptide gene enhancer in B-cells inhibitor- alpha
KEAP-1: Kelch like ECH associated protein 1
LPS: Lipopolysaccharide
MCP-1: Monocyte chemoattractant protein-1
MDA: Malondialdehyde
MRP: Multidrug resistance-associated protein
NAC: N-acetylcysteine
NADPH: Nicotinamide adenine dinucleotide phosphate
NGS: Next-generation sequencing
NF-*κ*B: Nuclear factor kappa-light-chain-enhancer of activated B cells
NQO1: NADPH quinone oxidoreductase 1
Nrf2: NFE2-related nuclear factor 2
OxLDL: Oxidized low-density lipoprotein
p38MAPK: p38 mitogen activated protein kinase
PARP: Poly ADP ribose polymerase
PGE2: Prostaglandin E2
Pgp1: ATP binding cassette subfamily B member 1 (ABCB1)
PKC: Protein kinase C
PMA: Phorbol myristate acetate
Prx1: Peroxiredoxin1
PUFA: Polyunsaturated fatty acid
RLIP76: RalA binding protein 1(RALBP1)
ROS: Reactive oxygen species
SOD: Superoxide dismutase
TLR-4: Toll-like receptor 4
TNF-*α*: Tumor necrosis factor *α*
TPA: Tissue plasminogen activator
Trx: Thioredoxin
UPR: Unfolded protein response
*γ*-GCL: *γ*-Glutamate cysteine ligase.
